# The effects of changing dairy intake on trans and saturated fatty acid levels- results from a randomized controlled study

**DOI:** 10.1186/1475-2891-13-32

**Published:** 2014-04-03

**Authors:** Jocelyne R Benatar, Ralph AH Stewart

**Affiliations:** 1 Green Lane Cardiovascular Service, Auckland City Hospital, Auckland, New Zealand; 2 Cardiovascular Research Unit, Greenlane Cardiovascular Service, Auckland City Hospital, Auckland 1030, New Zealand

**Keywords:** Trans fatty acids, Saturated fatty acids, Dairy food, Vaccenic acid, Palmitelaidic acid, Randomized controlled study

## Abstract

**Background:**

Dairy food is an important natural source of saturated and trans fatty acids in the human diet. This study evaluates the effect of dietary advice to change dairy food intake on plasma fatty acid levels known to be present in milk in healthy volunteers.

**Methods:**

Twenty one samples of whole fat dairy milk were analyzed for fatty acids levels. Changes in levels of plasma phospholipid levels were evaluated in 180 healthy volunteers randomized to increase, not change or reduce dairy intake for one month. Fatty acids were measured by gas chromatography–mass spectrometry and levels are normalized to d-4 alanine.

**Results:**

The long chain fatty acids palmitic (13.4%), stearic (16.7%) and myristic (18.9%) acid were most common saturated fats in milk. Four trans fatty acids constituted 3.7% of the total milk fat content. Increased dairy food intake by 3.0 (± 1.2) serves/ day for 1 month was associated with small increases in plasma levels of myristic (+0.05, 95% confidence level-0.08 to 0.13, p = 0.07), pentadecanoic (+0.014, 95% confidence level -0.016 to 0.048, p = 0.02) and margaric acid (+0.02, -0.03 to 0.05, p = 0.03). There was no significant change in plasma levels of 4 saturated, 4 trans and 10 unsaturated fatty acids. Decreasing dairy food intake by 2.5 (± 1.2) serves per day was not associated with change in levels of any plasma fatty acid levels.

**Conclusion:**

Dietary advice to change dairy food has a minor effect on plasma fatty acid levels.

**Trial registration:**

ACTRN12612000574842.

## Background

A healthy diet is important to reduce the risk of cardiovascular disease [1] and diabetes [2]. In observational studies, high intake of saturated [3] and trans fatty acids [4] and low intake of polyunsaturated fats [5] is associated with increased cardiovascular risk. Studies also suggest that increased plasma levels of trans [6] and saturated [7] fatty acids are associated with increased risk of cardiovascular disease. Plasma levels of trans, odd-numbered saturated and polyunsaturated (n-3 and n-6) fatty acids reflect dietary intake as these are not endogenously synthesized [8]. The aim of dietary advice to reduce intake of foods with saturated fats and TFA is to reduce plasma levels of ‘harmful’ fats and increase levels of ‘beneficial’ polyunsaturated fats.

Dairy food is a highly complex food comprised of constituents thought to be both harmful and beneficial for cardiovascular health. It is the most abundant source of animal fat in the diet including saturated fats and ‘naturally occurring’ ruminant trans fatty acids (rTFA) [8]. It is rich in in long chain saturated fatty acids such as myristic and palmitic acids; thought to be harmful for cardiovascular health [9]. However, dairy food also contains unsaturated, short and medium chain saturated fatty acids thought to be beneficial for heart health [10,11]. Whilst industrial trans fatty acids are considered harmful, the effects of trans fatty acids from dairy on cardiovascular health is less clear [12]. Some studies suggest rTFA may be beneficial to health [13] but others suggest a neutral effect [14,15]. These assumptions are based on the premise that certain trans fatty acids, like vaccenic acid, are exclusively from dairy [16], and others like elaidic acid are exclusively industrial [17].

This inconsistent data on the health effects of dairy [18-20] has led to confusing messages for consumers. Cardiovascular guidelines recommend avoidance of dairy fat [18,21] but others recommend at least 3–4 servings of dairy food per day [19].

No randomized studies have assessed the effects of changing dairy food intake in the real world on rTFA, saturated and polyunsaturated fatty acid levels. Consumers change intake of whole food rather than a specific fat. This study is designed to see whether changes in whole food result in significant changes in fatty acid levels. In this study, we analyzed the composition of fatty acid in dairy food in New Zealand. We then evaluated the effects of dietary advice to increase, decrease or not change daily dairy food intake for one month on plasma fatty acid levels in a randomized clinical trial with180 healthy volunteers.

## Methods

### Randomized study

A more detailed description of the randomized study , and effects of dietary intervention on cardio-metabolic risk factors have been reported previously [20]. Healthy volunteers (n = 180) living in Auckland, New Zealand regularly consuming dairy and who were willing to modify dairy intake for one month were recruited by advertisement from February 2011 to September 2011. Exclusion criteria included inability to tolerate dairy food, known diabetes, cardiovascular disease, inflammatory conditions, currently taking any lipid or glucose modifying medication and age ≤18 years. Ethics approval was obtained from the Northern X Ethics Committee and all participants provided written informed consent.

Participants were instructed to fast for 10 hours prior to clinic visits. Blood samples were collected in ethylene-diamine-tetra acetic acid tubes on all participants at baseline and after one month. Plasma was separated in a 4°Celsius centrifuge within 20 minutes of the blood draw and stored at -80°Celsius in nunc tubes until analysis.

Participants were randomized by a computer generated randomization algorithm to one of three possible arms; increased dairy (n = 60), reduced dairy (n = 60) or no change (n = 60) for 1 month. Participants were given advice on how to change dairy intake including written diet sheets. Participants randomized to increased dairy were asked to increase intake of full fat dairy food by more than three servings a day. Those asked to reduce dairy food were asked to eliminate dairy food and red meat as much as possible from the diet. This was to ensure that participants did not increase uptake of alternative sources of ruminant trans fatty acids. A follow-up assessment was arranged for one month. The National Cancer Institute Diet History Questionnaire [22], a validated food frequency questionnaire, was used to assess all dairy and red meat intake during the preceding 3 days at baseline and 1 month.

### Milk samples

Seven brands of whole (3.3% fat) cow’s milk were purchased in April 2011. These included homogenised, non-homogenised, organic and non-organic brands. Three samples of each brand were collected (total n = 21), decanted into nunc tubes and stored at -800 Celsius until analysis. Fasting blood samples were taken in ethylene-diamine-tetra acetic acid tubes. Plasma was separated and stored at -70°Celsius until analysis.

### Analysis of milk and plasma

Phospholipids analysis of milk and plasma was performed at the School of Biological Science, Auckland University. Total lipids were extracted with AR Methanol and fatty acid methyl esters formed by transmethylation. Fatty composition was assessed by gas chromatography–mass spectrometry (Hewlett Packard 6890 Gas Chromatograph with an SGE BPX70 column and a Flame Ionisation Detector. Peaks were normalised to d4-analine.

The coefficient of variation for each fatty acids at this laboratory is added to Table 1.

**Table 1:** Fatty acids in milk and in human plasma at baseline

	**Levels in whole fat milk N = 21**	**Coefficient of variation (%) milk**	**Plasma levels in humans at baseline**	**Coefficient of variation (%) plasma**
**Trans fatty acids**				
Fumaric acid (C4:1n2t)	0.059 (0.004)	0.071	ND	ND
Palmitelaidic acid(C16:1n7t)	0.007 (0.009)	1.292	0.022 (0.031)	1.593
Vaccenic acid (C18:1n7t)	0.334 (0.183)	0.551	0.458 (0.210)	0.465
Myristelaidic acid (C17:1n9t)	0.041 (0.006)	0.153	0.073 (0.313)	4.012
**Saturated fats**				
Caprylic acid (C8:0)	0.791 (0.213)	0.265	0.005 (0.003)	0.583
Capric acid (C10:0)	1.293 (0.347)	0.248	0.015 (0.019)	1.44
Lauric acid (C12:0)	1.520 (0.474)	0.322	0.034 (0.054)	1.633
Tridecylic acid (C13:0)	0.018 (0.003)	0.136	ND	ND
Myristic acid (C14:0)	1.996 (0.345)	0.179	0.262 (0.131)	0.476
Pentadecanoic acid (C15:0)	0.167 (0.088)	0.532	0.084 (0.032)	0.374
Palmitic acid (C16:0)	1.767 (0.265)	0.153	2.221 (0.635)	0.301
Margaric acid (C17:0)	0.122 (0.014)	0.824	0.160 (0.077)	0.534
Stearic acid (C18:0)	1.415 (0.137)	0.099	1.966 (0.548)	0.336
Arachidic acid (C20:0)	0.016 (0.002)	0.133	0.013 (0.004)	3.587
Lignoceric acid (C23:0)	0.006 (0.001)	0.224	ND	ND
**Polyunsaturated fats**				
Eicosatetraenoic acid (C20:4n8c,11c,14c,17c)	0.018 (0.003)	0.142	0.231 (0.868)	0.73
Linoleic acid (C18:2n9c,12c)	0.160 (0.012)	0.615	0.789 (0.373)	0.564
gamma-Linolenic acid (C18:3n6c,9c,12c)	0.176 (0.038)	0.222	0.076 (0.097)	1.230
Eicosadienoic acid (C20:2n11c,14c)	0.002 (0.002)	0.412	0.041 (0.017)	0.489
Arachidonic acid (C20:4n5c,8c,11c,14c)	0.020 (0.006)	0.285	0.556 (0.146)	0.283
Cervonic acid (C22:6n4c,7c,10c,13c,16c,19c)	0.024 (0.004)	0.283	0.381(0.132)	0.374
**Monounsaturated fats**				
Myristoleic acid (C14:1n9c)	0.12 (0.04)	0.33	ND	ND
Palmitoleic acid (C16:1n9)*	0.106 (0.026)	0.18	0.081(0.055)	0.53
Oleic acid (C18:1n9c)*	0.898 (0.085)	0.10	0.69(0.21)	0.32
Gondoic acid (C20:1n11c)*	0.004 (0.004)	0.78	ND	ND

Units are levels normalized to d4- alanine. Results are mean (standard deviation). The coefficient of variation for each plasma fatty acid is also listed.

ND = not detected.

*Monounsaturated fatty acids.

### Definitions

The United States Department of Agriculture criteria [23] were used to define serving size. For example one serving size was equivalent to 250 ml 3% milk, 250 ml yogurt, 500 ml 1.5% milk or 1/3 cup cheddar cheese. The total dairy fat content ingested in g/day was calculated based on the reported intakes of each food, and the fat content from manufacturers’ labels [24].

Saturated fatty acids were defined as short chain (aliphatic tails of2 < 6 carbons), medium chain (aliphatic tails of 6–12 carbons), long chain (aliphatic tails of 13–21 carbons), and very long chain (aliphatic tails of >21 carbons) [25].

### Statistical analysis

Baseline and one month visit levels of fatty acids and change in these measurements between baseline and one month were summarized as median and interquartile range. A 30% difference in vaccenic acid levels was expected to be seen with the intervention. Sixty in each group was estimated to give 80% power to detect a treatment difference with a two-sided 0.05 significance level, if the true difference in between the treatments is 1.5.

Analysis was done only on those participants who completed the study, as no measurable outcomes were available on those who did not return at one month. Baseline characteristics and scores were compared across the three arms using the analysis of variance (ANOVA). Analysis of covariance (ANCOVA) was used to determine if there were significant differences in fatty acid values between the three dairy groups [26]. ANCOVA was conducted on the follow up values adjusting for the baselines values. For the few variables that did not meet the ANCOVA assumptions [27], ANOVA was used to determine significant differences in follow-up values between the three dairy groups. Secondary analysis was performed to determine differences within randomized groups using ANOVA. Statistical analyses were performed with SAS software version 9.3 (SAS Institute, Cary, NC).

To adjust for multiplicity testing the Bonferroni method was used [28].

### Ethics approval

NTX/10/11/15 (Northern X ethics committee, Auckland, New Zealand).

## Results

### Fatty acids in milk

**Figure 1:**
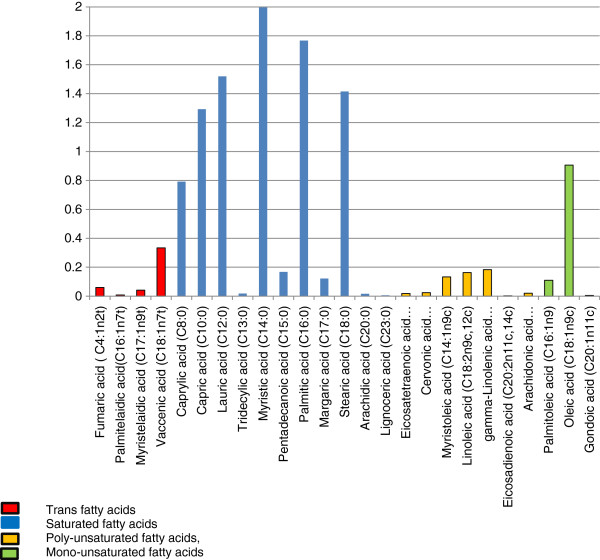
**Fatty acids found in New Zealand milk.** Plasma fatty acids are measured by mass spectrometry and normalized to d-4 alanine.

3.3% whole fat milk contained 90% saturated fats, 3.7% TFA and 5.3% unsaturated fats (Table 1). The most common saturated fats were the medium chain lauric acid 12.3 (SD 4.7)%, and long chain palmitic acid 14.3 (SD 5.8)% and myristic acid 16.1 (SD 3.5)%. Vaccenic acid was the most plentiful rTFA (2.8% of milk fat), and oleic acid is the most common unsaturated fat (Figure 1).

### Randomized study

176 of 180 randomized participants completed the study. Baseline characteristic are presented in Table 2. The mean age of the population was 47 (IQR: 38–55) years and 70% of the participants were female. The average body mass index was 24.5 (SD 4.0) and participants were normotensive with an average blood pressure (BP) 110/70 (SD10/8) mmHg. There were no significant differences in baseline characteristics by dietary group.

**Table 2:** Baseline characteristics according to randomized group

	**Reduced dairy intake**	**Same dairy intake**	**Increased dairy intake**
N	60	60	60
Female (%)	62	66	63
Age (years)	48.6 (12.0)	45.3 (12.4)	46.3 (10.5)
Heart rate (Beats per minute)	63.7 (8.2)	63.0 (9.1)	64.1 (8.6)
Systolic blood pressure	116.6 (12)	114.2 (10)	113.9 (11)
Diastolic blood pressure	70.3 (9)	70.0 (8)	70.2 (8)
Waist circumference (cm)	84.2 (12.3)	83.2 (12.6)	84.3 (9.8)
Hip circumference(cm)	100.1 (8.8)	100.8 (10.1)	101.7 (7.1)
BMI	24.2 (4.0)	24.8 (4.0)	24.6 (4.1)
TC (mmol/L)	5.31 (0.97)	5.13 (0.98)	5.15 (0.93)
HDL (mmol/L)	1.70 (0.42)	1.68 (0.47)	1.68 (0.48)
LDL (mmol/L)	3.12 (0.88)	2.97 (0.86)	2.98 (0.78)
TG (mmol/L)	1.12 (0.81)	1.12 (0.72)	1.04 (0.64)
Insulin resistance (HOMA)	1.38 (0.82)	1.43 (1.08)	1.47 (1.1)

Results are mean (standard deviation) and P > 0.03 for all.

Change in dairy food intake assessed by the FFQ across all three groups was significant (p < 0.001). For the increased dairy food diet this was +3.0 (1.2) serves/day, p < 0.001, no change was -0.6 (0.2) serves/day, p = 0.78 and for decreased dairy diet -2.5 (1.2) serves/day, p < 0.001. The total difference between those asked to increased and reduced dairy food intake was 5.5 (SD1.4) serves per day, p < 0.001. Change in dairy fat for the reduced dairy diet was -10.4 (10.1) g/day, no change was -3.4 (7.9) g/day, and increased dairy was +12.5 (15.7) g/day, p < 0.001. There was no significant change in intake of food from ruminant sources such as meat or goat milk and cheese by randomized group.

**Figure 2:**
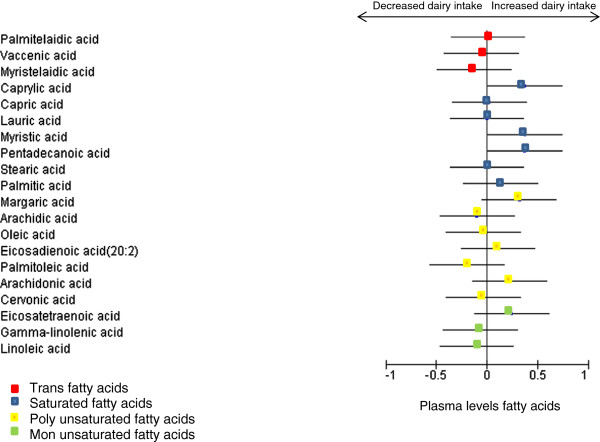
**Change in plasma levels of fatty acids with a high dairy compared to a low dairy food diet.** Results are expressed as a standard mean difference. This graph shows that increasing dairy food intake compared to the control group (no change) relatively increases plasma levels of caprylic, myristic, pentadecanoic and margaric acid, but has no effect on other fatty acids. However when correcting for multiplicity testing; only the change in pentadecanoic acid remained significantly different between high and low dairy diets.

The effect of changing dairy food intake on plasma fatty acid levels is displayed in Tables 3 and 4. There was no significant change in TFA between randomized groups. In those randomized to decrease dairy food intake, there was no significant change in fatty acid levels compared to the control group. In those randomized to increase dairy food intake, plasma levels of pentadecanoic (p = 0.02) and margaric acid (p = 0.03) increased compared to the control group. When comparison was made between increased and decreased dairy food intake (Figure 2), there was an increase in caprylic, myristic, pentadecanoic and margaric acid. Other long chain and medium chain saturated fats did not change significantly. After adjustment for multiple testing, change in pentadecanoic acid levels between increased and reduced dairy intake remained significant (p = 0.02). However, change in other fatty acid levels was no longer significant.

**Table 3:** The difference across the three dairy groups using ANCOVA

**Trans fatty acid**	**Reduced dairy intake**	**Same dairy intake**	**Increased dairy intake**	**Across all three groups P**	**Reduced vs. same P**	**Same vs. increased P**
*Vaccenic acid*						
Baseline	0.50	0.51	0.51			
	(0.32 to 0.63)	(0.36 to 0.61)	(0.37 to 0.61)			
1 month	0.54	0.49	0.50			
	(0.40 to 0.64)	(0.36 to 0.60)	(0.36 to 0.68)			
Change	0.05	-0.03	-0.04	0.74	0.24	0.31
	(-0.14 to 0.16)	(-0.23 to 0.26)	(-0.22 to 0.30)			
*Myristelaidic acid*						
Baseline	0.018	0.018	0.018			
	(0.015 to 0.024)	(0.013 to 0.023)	(0.015 to 0.023)			
1 month	0.019	0.018	0.018			
	(0.015 to 0.024)	(0.015 to 0.023)	(0.015 to 0.023)			
Change	0.001	0.001	0.001	0.79	0.91	0.26
	(-0.003 to 0.005)	(-0.004 to 0.005)	(-0.004 to 0.005)			
**Saturated Fatty Acid**						
*Myristic acid*						
Baseline	0.22	0.24	0.25			
	(0.17 to 0.30)	(0.18 to 0.32)	(0.19 to 0.33)			
1 month	0.25	0.24	0.29			
	(0.19 to 0.33)	(0.203 to 0.32)	(0.22 to 0.36)			
Change	0.01	0.01	0.05	0.39	1.00	0.07
	(-0.05 to 0.1)	(-0.08 to 0.09)	(-0.08 to 0.13)			
*Palmitic Acid*						
Baseline	2.33	2.32	2.20			
	(1.88 to 2.75)	(1.97 to 2.70)	(1.96 to 2.64)			
1 month	2.43	2.34	2.51			
	(2.09 to 2.89)	(1.97 to 2.91)	(2.06 to 2.93)			
Change	-0.52	0.42	1.37	0.79	0.47	0.42
	(-0.51 to 0.90)	(-0.47 to 0.62)	(-0.41 to 9.37)			
*Stearic acid*						
Baseline	1.98	1.98	1.97			
	(1.77 to 2.21)	(1.84 to 2.15)	(1.76 to 2.24)			
1 month	2.12	2.15	2.12			
	(1.88 to 2.35)	(1.91to 2.39)	(1.83 to 2.4)			
Change	+0.05	+0.17	+0.13	0.84	0.56	0.41
	(-0.27 to 0.48)	(-0.10 to 0.52)	(-0.32 to 0.56)			
*Pentadecanoic acid*						
Baseline	0.084	0.084	0.085			
	(0.072 to 0.101)	(0.076 to 0.120)	(0.070 to 0.101)			
1 month	0.079	0.085	0.098			
	(0.054 to 0.088)	(0.068 to 0.104)	(0.078 to 0.120)			
Change	-0.006	-0.00	+0.014	0.11	0.33	0.02
	(-0.015 to 0.00)	(-0.015 to 0.017)	(-0.016 to 0.048)			
*Margaric acid*						
Baseline	0.17	0.17	0.16			
	( 0.12 to 0.22)	( 0.12 to 0.21)	( 0.09 to 0.21)			
1 month	0.15	0.17	0.18			
	( 0.08 to 0.19)	( 0.08 to 0.18)	(0.14 to 0.24)			
Change	-0.02	-0.001	+0.02	0.95	0.54	0.03
	( -0.09 to 0.03)	( -0.08 to 0.02)	(-0.03 to 0.05)			

ANCOVA was used unless assumptions were not met. ANOVA was used for all others (Table 4) Fatty acids are standardized to d4- alanine and presented as median (interquartile range).

**Table 4:** The difference across the three dairy groups using ANOVA for those fats who did not meet the assumptions for ANCOVA

**Trans fatty acid**	**Reduced dairy intake**	**Same dairy intake**	**Increased dairy intake**	**P value**
*Palmitelaidic acid*				
Baseline	0.033	0.04	0.02	
	(0.00 to 0.45)	(0.00 to 0.43)	(0.00 to 0.32)	
1 month	0.049	0.039	0.034	
	(0.00 to 0.50)	(0.00 to 0.49)	(0.01 to 0.39)	
Change	0.00	0.00	0.00	0.96
	(-0.09 to 0.09)	(-0.27 to 0.22)	(-0.07 to 0.14)	
*Linoelaidic acid*				
Baseline	0.000	0.000	0.000	
	(0.000 to 0.0296)	(0.000 to 0.026)	(0.001 to 0.018)	
1 month	0.000	0.000	0.000	
	(0.000 to 0.0233)	(0.000 to 0.0274)	(0.000 to 0.033)	
Change	0.000	0.000	0.000	0.53
	(-0.010 to 0.000)	(-0.014 to 0.016)	(0.000 to 0.018)	
**Saturated fats**				
*Lauric acid*				
Baseline	0.025	0.026	0.025	
	(0.000 to 0.039)	(0.000 to 0.043)	(0.000 to 0.042)	
1 month	0.030	0.026	0.036	
	(0.000 to 0.056)	(0.000 to 0.041)	(0.025 to 0.052)	
Change	0.00	0.000	0.012	0.12
	(-0.011 to 0.027)	(-0.022 to 0.024)	(-0.005 to 0.024)	

There was no significant change in total saturated fats for increased dairy diet +13.1 (95% confidence interval (CI) -1.2 to + 25.3)%, same dairy diet +2.4 (95% CI -9.2 to +11.3)% or decreased dairy diet +3.5 (95% CI -5.5 to +11.6)%. The FFQ indicated that participants randomized to reduce dairy food intake switched to rice, almond and soy milk products.

Change within randomized groups was then analyzed. There was no significant change in total rTFA for increased dairy diet -5.5% (95% CI -15.4 to +10.2 )%, same dairy diet -9.1(95% CI -20 to +1.2)%, and decreased dairy diet +10.1 (95% CI -2.4 to +22.5)%. Levels of the individual trans fatty acid isomers, vaccenic acid, palmitelaidic acid and myristelaidic acid also did not change. Pentadecanoic (15:0), myristic (14:0) and margaric (heptadecanoic (17:0)) acid increased with increased dairy intake (p = 0.02 and 0.03 respectively); however, there was no significant change for reduced dairy intake.

**Figure 3:**
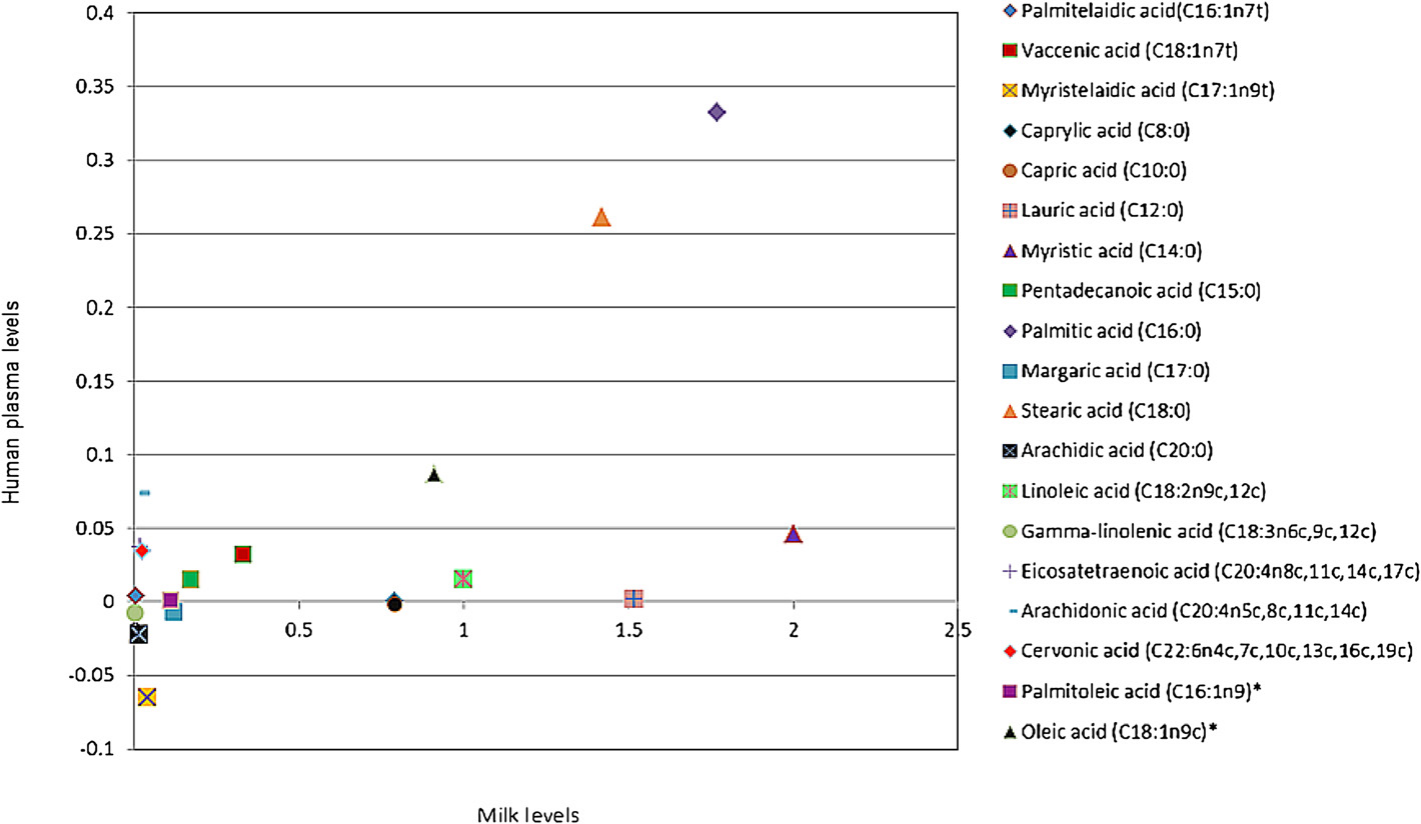
**Correlation between fatty acid levels in milk and change in plasma fatty acid levels in those participants asked to increase dairy food intake.** Results are mean levels and are normalized to d4 alanine.

**Figure 4:**
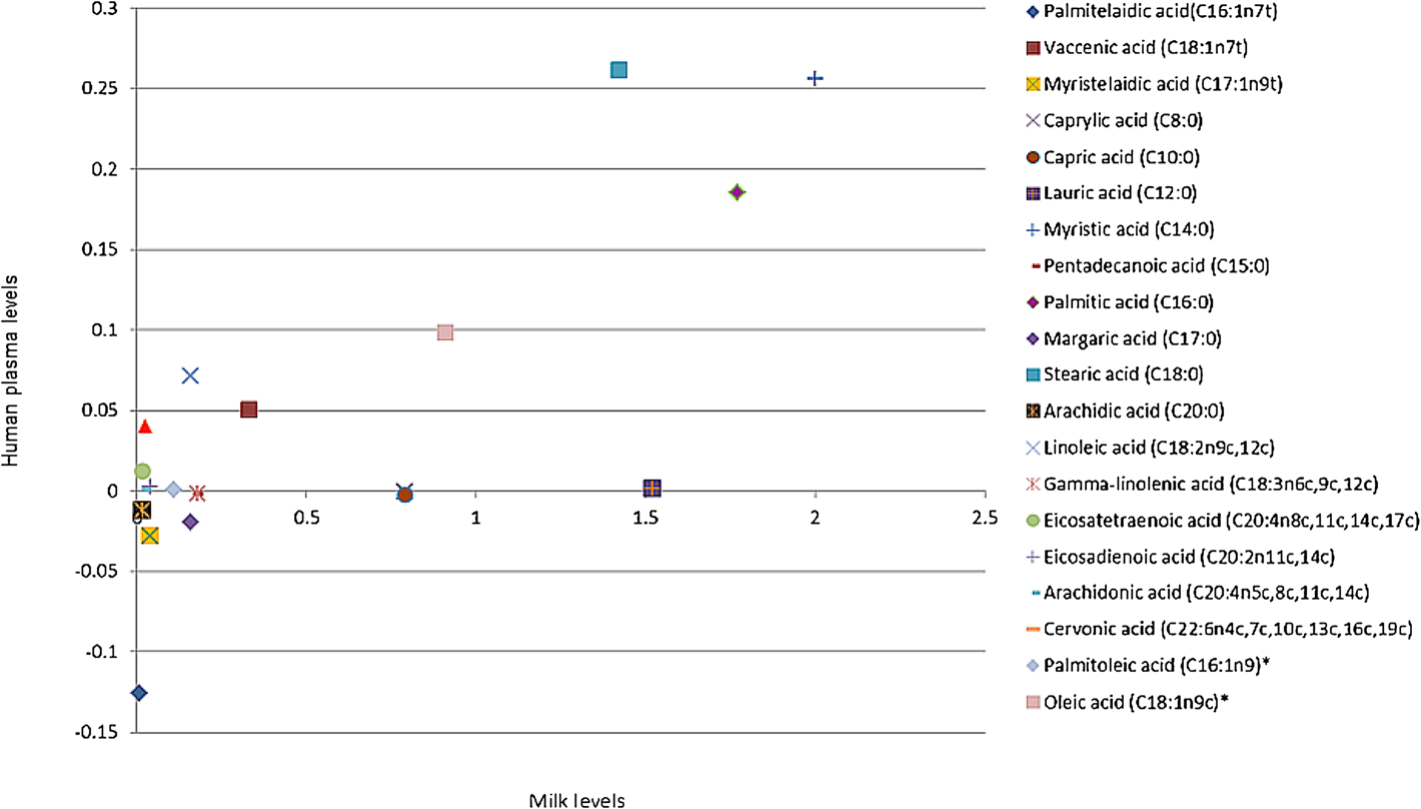
**Correlation between fatty acid levels in milk and change in plasma fatty acid levels in those participants asked to decrease dairy food intake.** Results are mean levels and are normalized to d4 alanine.

Figure 3 shows associations between levels of milk fatty acids and effects of increasing dairy food intake on plasma levels of fatty acids. Figure 4 shows associations between levels of milk fatty acids and effects of reducing dairy food intake on plasma levels of fatty acids. Change in plasma levels of fatty acid isomers did not correlate with the amount of that fatty acid in dairy food. For example, myristic acid and palmitic acid were the most plentiful saturated fats in milk, but changing dairy food intake did not correlate with change in plasma levels.

## Discussion

Dairy food is the richest natural source of ‘harmful’ fats like long chain saturated and trans fatty acid in the diet [29]. Changing dietary intake should significantly affect plasma levels of these fats, especially long chain fatty acids, and rTFA thought to be almost exclusively from dairy food. In this study cow’s milk was analyzed to assess fatty acids present in dairy food. Fatty acid composition in milk is mainly dependent on the feed and seasonality [30] and varies between countries. It was therefore essential to confirm fatty acids levels concurrent with the randomized study to allow for interpretation of study findings. Milk was considered the most useful dairy food to analyze as participants increased dairy food intake mainly by increasing intake of whole fat milk, yogurt and ice cream rather than cheese.

Intake of dairy food significantly changed for all those randomized to increase and decrease dairy food intake, with those that increased dairy food consuming on average 5.5 serves per day more than those that decreased dairy food intake. However, whilst there was a small increase in plasma levels of myristic, pentadecanoic acid and margaric acid with increased dairy consumption, plasma levels of most fatty acids present in milk did not change with diet. After multiplicity testing, only pentadecanoic acid changed significantly.

Dietary guidelines recommend consumption of low fat dairy food to reduce intake of long chain fatty acids like myristic and palmitic acid that are associated with increased cardiovascular risk [31], and stearic acid that is associated with reduced HDL cholesterol [32]. However the effects of pentadecanoic acid, which increased in those asked to increase dairy food intake, are not known. Some studies have suggested that margaric acid and pentadecanoic acid are markers of dairy food intake [33], however this study suggests that in the real world, large changes in dairy food intake marginally changes fatty acids. Moreover, reducing dairy food intake had no effect on plasma levels of these fats suggesting that these may be present in other food sources. Participants increased uptake of food that are good sources of saturated fats such as foods with palm and coconut oils (such as table spreads, processed foods) and almond milk. Ultimately, separating the health effects of these fatty acids is difficult to justify when giving dietary advice, as they are highly correlated because they are found in the same food (e.g., beef and dairy products).

Similarly, rTFA did not change significantly between groups. Dietary advice to reduce dairy food intake in patients with cardiovascular disease is not aimed at reducing plasma rTFA levels. Consumption of rTFA is thought to be too low to have biological effects [34] and some studies suggest that vaccenic acid may not be harmful for human health [13]. Previous studies have identified palmitelaidic acid as exclusively from dairy food [35,36]; however, this study suggests that these assumptions may be misplaced.

It is possible in persons who decreased dairy food, rTFA from other sources increased. Oils containing precursors to vaccenic and palmitelaidic acid are increasingly used for processing and cooking food. Increased heat or pressure during cooking can increase rTFA levels in these oils [37]. Vaccenic acid is found in dairy free processed foods such as takeaway chips in the United Kingdom [38]. Dairy is also added to products like margarines, often assumed to be dairy- free. When these are heated, TFA levels can significantly be increased [37]. A less likely possibility is that humans are able to catalyze a reaction to convert small amounts of cis to TFA. Whilst humans are able to convert vaccenic acid to conjugated linoleic acid by delta-9 desaturase in the liver [39,40], bioconversion of cis to trans fatty acids has not been demonstrated.

Changing dairy food intake for one month could be too short to affect plasma TFA levels. However, in feeding studies changes in plasma TFA occur within 2–3 weeks [41,42], and other dairy fatty acids like pentadecanoic and margaric acid [33], changed with a change in dairy intake within 3 weeks. There was a large difference in dairy food intake within and between groups. In contrast to some observational studies [35,36], no correlation was observed between vaccenic acid and palmitelaidic acid either at baseline or 1 month (r = 0.053 and 0.089 respectively) suggesting they are not exclusively from the same source.

### Limitations

It is possible participants did not adhere to the randomized diet. However, food frequency questionnaires suggested good adherence and that consumption of other sources of TFA such as processed food, and goat’s milk or meat were not increased.

Food frequency questionnaires may not accurately reflect dietary intake [43,44]. Studies comparing food frequency measures with repeated dietary recalls generally show correlations of the order of 0.4–0.7 [45].

## Conclusion

Dietary advice to change the intake of dairy food does not significantly change plasma fatty acid levels including ruminant trans fatty acids. Dietary advice may need to focus on total food patterns rather than individual food groups to affect plasma fatty acid levels.

## Abbreviations

SD: Standard deviation; CI: Confidence interval; TFA: Tran fatty acid(s); rTFA: Ruminant trans fatty acid(s); ANOVA: Analysis of variance; ANCOVA: Analysis of covariance; IQR: Interquartile range; FFQ: Food frequency questionnaire.

## Competing interests

All authors have completed the ICMJE uniform disclosure form at http://www.icmje.org/coi_disclosure.pdf (available on request from the corresponding author) and declare: no support from any organisation for the submitted work; no financial relationships with any organisations that might have an interest in the submitted work in the previous three years; and no other relationships or activities that could appear to have influenced the submitted work.

## Authors’ contribution

JB conceived and designed the study, carried out the study, performed the statistical analysis and wrote the first draft of the manuscript. RS conceived the study and revised the manuscript critically for intellectual content. All authors read and approved the final manuscript.
